# Implementation of Sensing and Actuation Capabilities for IoT Devices Using oneM2M Platforms

**DOI:** 10.3390/s19204567

**Published:** 2019-10-21

**Authors:** Jaeseok Yun, Il-Yeup Ahn, JaeSeung Song, Jaeho Kim

**Affiliations:** 1 Department of Internet of Things, Soonchunhyang University, Asan 31538, Korea; 2 Intelligent IoT Research Center, Korea Electronics Technology Institute, Seongnam 13509, Korea; iyahn@keti.re.kr; 3 Department of Information Security, Sejong University, Seoul 05006, Korea; jssong@sejong.ac.kr; 4 Intelligent IoT Research Center, Korea Electronics Technology Institute, Seongnam 13509, Korea; jhkim@keti.re.kr

**Keywords:** IoT devices, sensing, actuation, oneM2M standard, open source

## Abstract

In this paper, we present an implementation work of sensing and actuation capabilities for IoT devices using the oneM2M standard-based platforms. We mainly focus on the heterogeneity of the hardware interfaces employed in IoT devices. For IoT devices (i.e., Internet-connected embedded systems) to perform sensing and actuation capabilities in a standardized manner, a well-designed middleware solution will be a crucial part of IoT platform. Accordingly, we propose an oneM2M standard-based IoT platform (called nCube) incorporated with a set of tiny middleware programs (called TAS) responsible for translating sensing values and actuation commands into oneM2M-defined resources accessible in Web-based applications. All the source codes for the oneM2M middleware platform and smartphone application are available for free in the GitHub repositories. The full details on the implementation work and open-source contributions are described.

## 1. Introduction

Since the term was coined by Kevin Ashton in 1999, the Internet-of-Things (IoT) has been an obvious trend and has crept into our everyday life, for example, appearing as Internet-connected home appliances such as a WiFi-enabled, programmable thermostats or LED light bulbs embedded with a ZigBee radio chip. Such home appliances are directly, or via a gateway, connected to the Internet so that end-users can access them remotely from anywhere at any time perhaps mostly through smartphones; for example, set the desired temperature of rooms while driving back to home from workplace.

The advances in IoT are changing our home to a ’connected home’ where home appliances and utilities connected each other can share the information about the changes in their status and surroundings, and thus provide home occupants with smart services in a proactive and intelligent manner [1]. Also, in the manufacturing sector IoT is considered as a key to the ’smart factory’ where all things in the factory itself as well as throughout the supply chain are fully connected and thus almost real-time data analytics and insights could be generated, helping manufacturers adapt their facilities and assets accordingly, maintain workforce productivity efficiently, and manage their supply chain proactively [2].

A key requirement of realizing the connected world is to ensure interoperability among different connected devices, i.e., enable all different types of things to talk to each other. However, it seems a challenging task to make different types of devices interoperable because they will have a wide variety of heterogeneous hardware systems, for example in case of a home, ranging from the front door (e.g., near field communication-based door locks) to the basement (e.g., programmable thermostats linked to its heating/cooling system that helps avoid peak-time energy use). Indeed, these systems will rely on different network protocol stacks and their proprietary service layer platforms.

Middleware solutions could play a pivotal role in bridging the highly fragmented market for IoT industry. Specifically, they can help interconnected devices communicate and “understand” each other even though each speaks its own language [3]. Therefore, although it might be a challenging task to achieve a universal standard for IoT, it would be very helpful for individual programmers and numerous organizations to provide a well-designed middleware solution that can interpret a wide variety of low-level hardware signals (e.g., digital input and outputs) to an Internet-accessible resource (e.g., Web object) based on widely used IoT standards, which echoes our motivation for this research.

The main contribution of this paper are three fold. First, we develop an IoT software platform (called nCube) complying with the oneM2M, a well-known IoT standard, which works as a middleware for IoT devices to support various different hardware interfaces including digital input and output, UART (universal asynchronous receiver/transmitter), SPI (serial peripheral interface), I^2^C (inter-integrated circuit). Second, we also develop an Android application working with the nCube-equipped IoT devices via oneM2M-defined Web APIs (application programmable interfaces) that enable to access and interact with sensors and actuators of the IoT devices. Finally, all source codes for the developed software are shared via GitHub for free in order for many other developers to download and utilize them in building new IoT products in the fragmented ecosystem of IoT industry.

The remaining organization of the paper is as follows. Section 2 summarizes the requirements for IoT middleware and Section 3 presents various IoT middleware solutions and the interoperability issue in our IoT landscape. Section 4 introduces the oneM2M-based scenarios for sensing and actuation capabilities of IoT devices, and Section 4.2 demonstrates our oneM2M middleware platform and its architecture for IoT devices. In Section 5, we present the implementation work and open-source contribution. Section 6 discusses some limitations of our method and remaining challenges. Finally, Section 7 offers concluding remarks.

## 2. Related Works

IoT envisions everyday objects and industrial products to be connected together, sharing their information over the Internet, working and interacting in a cooperative way, and thus building a *smart* environment. Many research works have been working on realizing IoT middleware for smart solutions in a variety of application domains. Brouwers et al. developed a pragmatic middleware framework for mobile phone sensing that provides features fine-grained user-level control to protect privacy of smartphone users [4]. The proposed middleware was implemented on the Android platform outlining an architecture featuring a publish and subscription framework and evaluated with a localization application. Silva et al. implemented an architecture for smart solutions based on IoT technologies, which consists of user interfaces, devices, and middleware [5]. They validated the architecture using the scenarios involved with a monitoring device of water and electricity consumption and LED-based user interfaces. Alvisi et al. developed a highly interoperable smart water metering solution using an interoperable wireless IoT middleware based on the Edge computing paradigm [6]. They developed smart metering devices using a Raspberry Pi, LTE module, and wireless radio modules including LoRa and M-Bus, increasing interoperability across heterogeneous wireless protocols. Benayache proposed a micro-service middleware for smart wireless sensor network-based IoT applications inspired from artificial neural network architecture, which allows dynamic service integration and solves the heterogeneity and interoperability problems [7]. Souza Cimino et al. introduced a middleware solution for integrating high performance computing characteristics into IoT systems to build user applications requiring both high computing performance and IoT features such as smart logistics and smart buildings [8]. They implemented a Java/Android based middleware solution and evaluate it with an increasing number of sensors and devices connected through several concurrency scenarios.

A great deal of effort has been devoted to clarify requirements for IoT middleware , including network protocol bridging [9,10], device and network management [11], resource searching [12], REST (representational state transfer) API [13], semantics [14,15], security [16], and support for cloud and fog computing [17,18,19,20]. Cruz et al. proposed an application layer gateway (called MiddleBridge) running on Java virtual machine, which translate widely used message protocols including constrained application layer protocol (CoAP), message queuing telemetry transport protocol (MQTT), data distribution service (DDS), and WebSocket to hypertext transfer protocol (HTTP) [9]. Bouloukakis et al. introduced a solution for the automated synthesis of lightweight protocol mediators by abstracting any IoT protocol with a data exchange API, allowing for middleware-layer protocol interoperability in the IoT [10]. Silva et al. proposed a new IoT management platform that supports important requirements for IoT networks and device management including security, context-aware, a standard model for message exchange, and friendly user interface [11]. Pattar et al. presented a comprehensive review of the existing search methods for the IoT resources and categorized them according to design principles and search approaches [12]. They highlights an IoT middleware as a key component of the search system, which supports scalability, heterogeneity and inteoperability. Cheng et al. introduced a lightweight IoT service mashup middleware based on REST-style architecture so as to build comprehensive and situational mashup applications in response to demands by the end users [13]. They illustrated the scenarios of IoT service mashups for coal mine safety monitoring and control and perform an evaluation study. Tao et al. presented a multi-layer cloud architectural model to enable effective and seamless interactions/interoperations on heterogeneous devices/services in IoT-based smart homes [14]. In particular, an ontology-based security service framework is proposed in order to provide security and privacy preservation in interactions and interoperations. Caballero et al. presented an ontology-defined middleware-agnostic approach for IoT architectures in order to allow the reusability and shareability of the execution flow among heterogeneous IoT devices by describing and executing the behavior of IoT devices using ontology-based technologies [15]. Ammar et al. presented a survey of the security of the IoT frameworks [16]. Stergiou et al. presented a survey of IoT and Cloud Computing with a focus on the security issues of both technologies [17]. Nguyen et al. reported the preliminary results of a systematic mapping study to support the deployment or orchestration at IoT device levels across IoT, edge, and cloud infrastructures [18]. Aazam et al. analyzed IoT middleware technologies in mobile and IoT service provisioning architecture under the concept of fog computing paradigm [19]. Farahzadi et al. presented a survey of IoT middleware that can address the problem of heterogeneity of different objects in terms of main features and characteristics [20]. The authors also introduced middleware appropriate for building platforms based on Cloud of Things (CoT) to enhance the large-scale IoT platforms.

## 3. IoT Middleware and oneM2M Standard

In particular, a few literature reviews over the past years summarized issues and challenges on requirements for IoT middleware by examining relevant articles [21]. Razaque et al. highlights the importance of middleware for the IoT, which allows massive amounts of heterogeneous devices to be connected by means of diverse networks and platform technologies, supporting interoperability across physical and virtual objects and enabling a wide range of applications and services [22]. They outlined a set of requirements for IoT middleware, including functional, non-functional, and architectural requirements and summarized a comprehensive review of the published middleware solutions against their proposed requirements. Ngu et al. conducted an extensive survey on the existing IoT middleware and categorized them into service-based, cloud-based, and actor-based middleware [23]. They also summarized the issues and challenges in developing an IoT middleware, including the heterogeneity of IoT divides, need to support the essential ingredients of composition, adaptability, and security aspects of an IoT system.

As many research and survey works described above, IoT middleware (sometimes called IoT platform) will be crucial to the development of IoT domains, and its requirements could be categorized into functional and non-functional aspects as described in [3]. Table 1 summarized the requirements.

Several standard development organizations have been working on developing IoT standards for solving the fragmentation of IoT landscape. Among them, the oneM2M initiative aims to develop standardized technical specifications considering a common horizontal M2M/IoT service layer platform across a number of industry verticals for globally applicable, access independent M2M/IoT services [24]. The oneM2M standard focuses on interoperability in service layers rather than protocol stacks in network and Internet layers, e.g., IEEE 802.15.4 and wireless long-range network. Accordingly, the oneM2M standard defines common service functions for IoT systems, including device registration and discovery, data management and repository, subscription and notification, semantics, security, etc., which support most of the requirements summarized in Table 1.

The oneM2M standard-based IoT platforms have shown its practical feasibility in developing home appliances [25], smart farm [26], smart office [27], medication [28], and semantic interworking in smart cities [29,30]. Recently, Ouedraogo et al. considered the quality of service (QoS) required by the IoT applications as scientific challenges, and then presented a solution consisting of the dynamic, autonomous and seamless deployment of QoS management mechanism integrated with an oneM2M standard-based IoT middleware [31]. Zhao et al. proposed an oneM2M-complaint stacked middleware to integrate open-source hardware and software projects for IoT development and research [32].

Interoperability in IoT would be achieved by speaking the same language (i.e., a universal standard like the oneM2M) [33] or bridging two different systems and translating their messages and protocols each another [34], which is the key role of IoT middleware. However, another important feature of IoT middleware, in particular for devices (i.e., embedded systems), is to provide an abstraction layer that masks the complex underlying hardware interfaces wired with sensors and actuators and instead exposes a set of useful APIs with which IoT applications can access their resources residing in IoT platforms. In this paper, we call this *sensing and actuation capabilities* for IoT middleware. Although the oneM2M-based IoT middleware platform considering such capabilities was proposed in the previous work [25], it was implemented as a Java program and has some limitations on fast and easy development compared to other programming languages (e.g., Node.js) supported by a lot of community-developed codes, providing our motivation into this research.

## 4. oneM2M Platform for Sensing and Actuation of IoT Devices

### 4.1. oneM2M-Based Scenarios for Sensing and Actuation Capabilities

**Figure 1:**
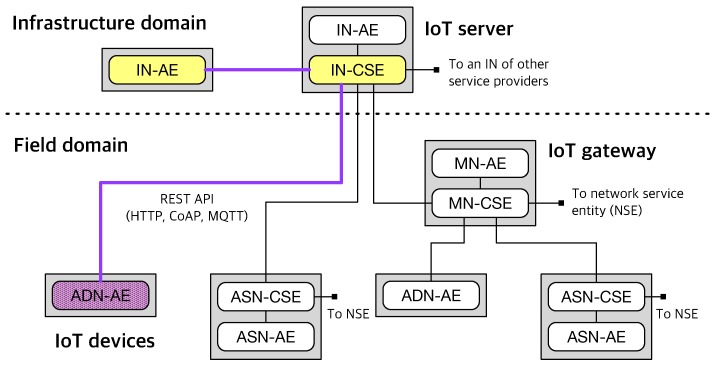
The oneM2M reference architecture.

The oneM2M standard supports resource-oriented architecture (ROA) and adopts a layered model as shown in Figure 1. Figure 1 illustrates the oneM2M reference architecture whose environments are divided into two domains: infrastructure domain where an infrastructure node (IN) locates and field domain where middle nodes (MNs), application service nodes (ASNs), and application dedicated nodes (ADNs) locate. IN, MN, ASN have to contain common service entities (CSEs) which provide common service functions (e.g., the functional requirements described in Table 1) to other CSEs or application entities (AEs) which provide application service logics (e.g., sensing and actuation). Networking services are provided by network service entities (NSEs) located in the underlying network service layer. More details are well described in the previously published literature [35].

**Figure 2:**
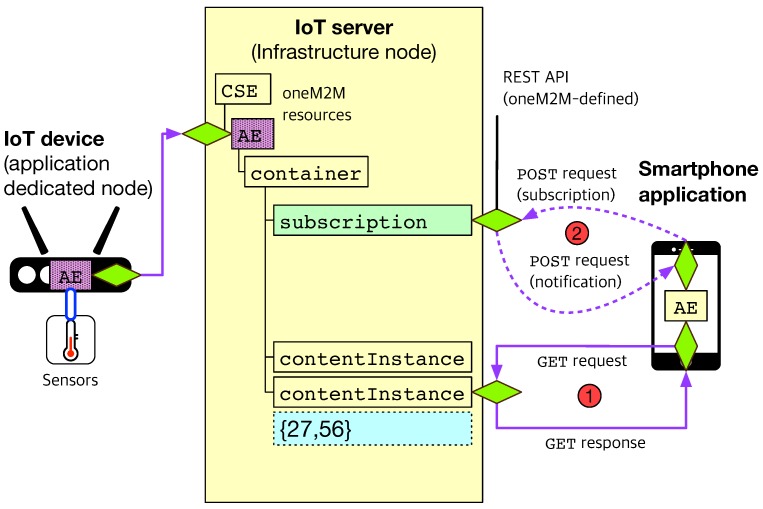
The proposed sensing scenario using oneM2M standard-based sensing capabilities.

**Figure 3:**
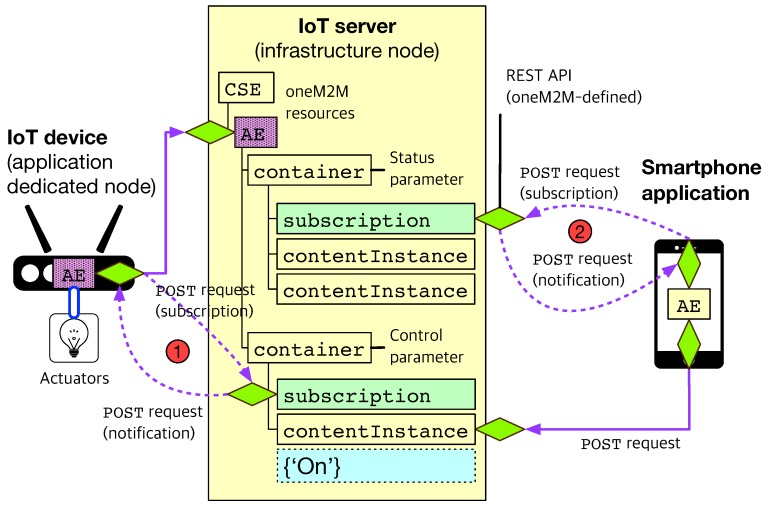
The proposed actuation scenario using oneM2M standard-based actuation capabilities.

In our work, we focus on the sensing and actuation capabilities for IoT devices, so three components are mainly considered from the oneM2M reference architecture as highlighted in Figure 1: IoT server (IN-CSE), IoT device (ADN-AE), smartphone application (IN-AE). We assume that all three components can talk to each other via oneM2M standard-defined REST APIs on HTTP, CoAP, or MQTT protocols. With this assumption, we can image sensing and actuation scenarios between IoT devices and smartphone applications as demonstrated in Figure 2 and Figure 3.

On the left side of Figure 2, the AE residing in the IoT device embedded with a temperature sensor is registered to the IoT server. In other words, an AE resource for the IoT device is created under the CSE resource of the IoT server. Subsequently, a container resource is created under the AE for the ‘thing’ connected to the IoT device, i.e., the temperature sensor. After that registration procedure, the IoT device will create new contentInstance resources under the container if needed (e.g., at a regular sensing interval), in which each contains the sensing value of the temperature sensor. The string example of {27,56} represents a temperature of 27° in Celsius and a relative humidity of 56%.

While the IoT device creates new contentInstance resources, the AE residing in the smartphone application on the right side of Figure 2 is able to get the sensing values via oneM2M standard-defined REST APIs supported by the IoT server.

It provides two types of methods: request/response and subscription/notification. In the first method (marked by the circled number of ‘1’), the smartphone AE sends a HTTP GET request to the IoT server with the URL (uniform resource locator) linked to the contentInstance resource it wants to get. If the request is asked with the appropriate privileges (called access control policy in the oneM2M standard), the IoT server sends back the smartphone AE the HTTP response containing the JSON (JavaScript object notation) or XML (extensible markup language) body, which stands for the temperature sensor’s sensing value.

The second method (marked by the circled number of ‘2’) can be accomplished by the common service function of the oneM2M platform, called ‘subscription/notification’. Since the smartphone AE creates a subscription resource under the container which it is interested in, the IoT server will notify its subscriber such as the smartphone AE of any events under the subscribed resource. Accordingly, the smartphone AE will be able to get alerts as soon as new contentInstance resources are created under the container (i.e., just after the IoT device measures new temperature values and creates its corresponding contentInstance resources under the container). All these subscription/notification procedures will be performed by the HTTP POST requests.

Figure 3 illustrates the actuation scenarios for IoT devices. On the left side of Figure 3, the AE residing in the IoT device embedded with a lightbulb is registered to the IoT server. Subsequently, the IoT device creates two container resources under the AE for the lightbulb, one for “light control” and the other for “status monitoring”. After that registration procedure, the IoT device will create a new subscription resource (marked by the circled number of ‘1’) in order to get alerts as soon as new contentInstance will be created under the container (i.e., to be notified of control messages triggered by other IoT applications). The string example of {‘n’} represents a semantic command of ‘turn the light on’.

On the right side of Figure 3, the smartphone AE can send the lightbulb incorporated into the IoT device a control command by creating a new contentInstance under the container for light control with the HTTP POST request. At the same time, the smartphone AE can create a subscription resource under the container for status monitoring. The subscription/notification function (marked by the circled number of ‘2’) is intended to get notified when the IoT device updates its new status, e.g., ‘the light is now on’. Actually, this status monitoring procedure can be optional, but it would be clearly helpful for the smartphone AE to monitor the change of status of the IoT device, for example, to clarify whether the IoT device performs the control command properly or not. Likewise the sensing scenario described above, the subscription/notification procedures for the actuation scenario will be performed by the HTTP POST requests defined in the oneM2M standard.

### 4.2. oneM2M Middleware Platform for IoT Devices

**Figure 4:**
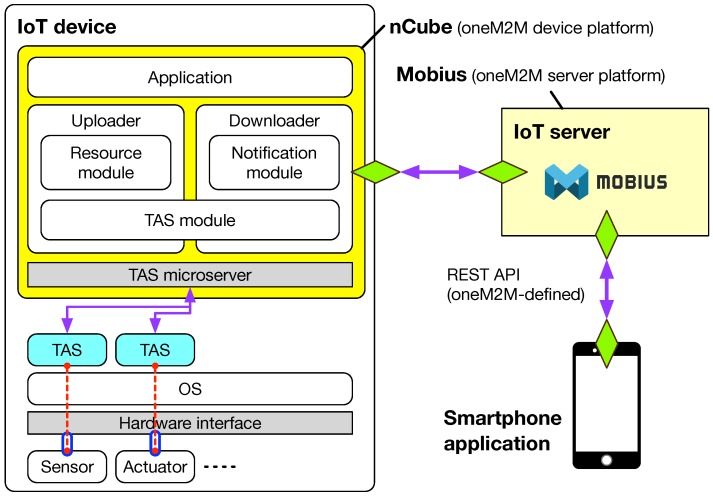
The architecture for the oneM2M middleware platform.

With the sensing and actuation scenarios with oneM2M-based sensing and actuation capabilities described above, we propose the architecture for the oneM2M middleware platform as shown in Figure 4. Here, we assume that the IoT server is supported by the oneM2M server platform, called the Mobius, described in the previous work [35].

The oneM2M device platform, called nCube, was originally presented in the previous work [25]. However, it was implemented as a Java program that requires the Java runtime environment (JRE) and thus has some limitations, in particular for event-driven input/output applications of resource-constrained IoT devices. In addition, from the point of view of fast and easy development, we need to adopt another Web-based framework supported by a lot of community-developed codes supporting a wide variety of hardware interfaces deployed in IoT devices. Accordingly, we propose a new version of the architecture for the oneM2M device platform, as shown in Figure 4.

The nCube consists of six modules: Application, Uploader, Downloader, Resource module, Notification module, and TAS module. In particular, a set of tiny software programs called ‘thing adaptation software (TAS)’ are responsible for translating the messages transmitted from sensors or to actuators of IoT devices through the corresponding hardware interfaces. In other words, a TAS works as a software glue (i.e., middleware) translating proprietary sensor data formats and actuator commands into oneM2M standard-defined resources or vice versa. Accordingly, the nCube can be referred to as an IoT middleware platform as described in [3]. Also, a TAS is considered as an interworking proxy entity (IPE) in the oneM2M standard offering interworking capabilities between the oneM2M system (i.e., IoT devices) and non-oneM2M system (i.e., sensors and actuators).

Among various hardware interfaces, we mainly considered two types of interfaces: input/output and communication, as summarized in Table 2. For input and output interfaces, we chose digital inputs and outputs. For supporting communication interfaces, we selected UART, SPI, and I^2^C. Analog inputs and outputs (i.e., analog-to-digital conversion and digital-to-analog conversion) could be supported by employing additional hardware, for example, MCP3008 analog-to-digital converter (ADC) with SPI communication interface. Because most of sensors and actuators used in widely deployed embedded systems can be covered by the hardware interfaces listed in Table 2, all we need to do is to develop the TAS program corresponding to each hardware interface.

## 5. Implementation and Open Sources

### 5.1. Software Environment and Hardware Platform

For realizing the proposed IoT middleware platform, we need to consider two important aspects: software environment and hardware platform appropriate to our implementation work, which shows the practical feasibility of the oneM2M standard-based sensing and actuation capabilities.

First, Node.js was chosen for the software environment because it provides a JavaScript runtime environment running real-time Web-based applications outside of a browser. Also, it can offer non-blocking, asynchronous, event-driven input/output models, which would be very efficient for resource-constrained IoT devices, e.g., single-thread machine frequently interacting with file systems, networks, and peripherals. In addition, Node.js developers can be supported by open-source communities (e.g., node package manager [36]) providing thousands of open-source libraries including those for hardware interfaces. This open-source-based contribution has proved to be very effective in development but also research works [37,38].

Second, the Raspberry Pi was chosen for our hardware platform because it offers all the hardware interfaces listed in Table 2 but also a lot of Node.js packages tested for the Raspberry Pi are available in open-source communities.

**Figure 5:**
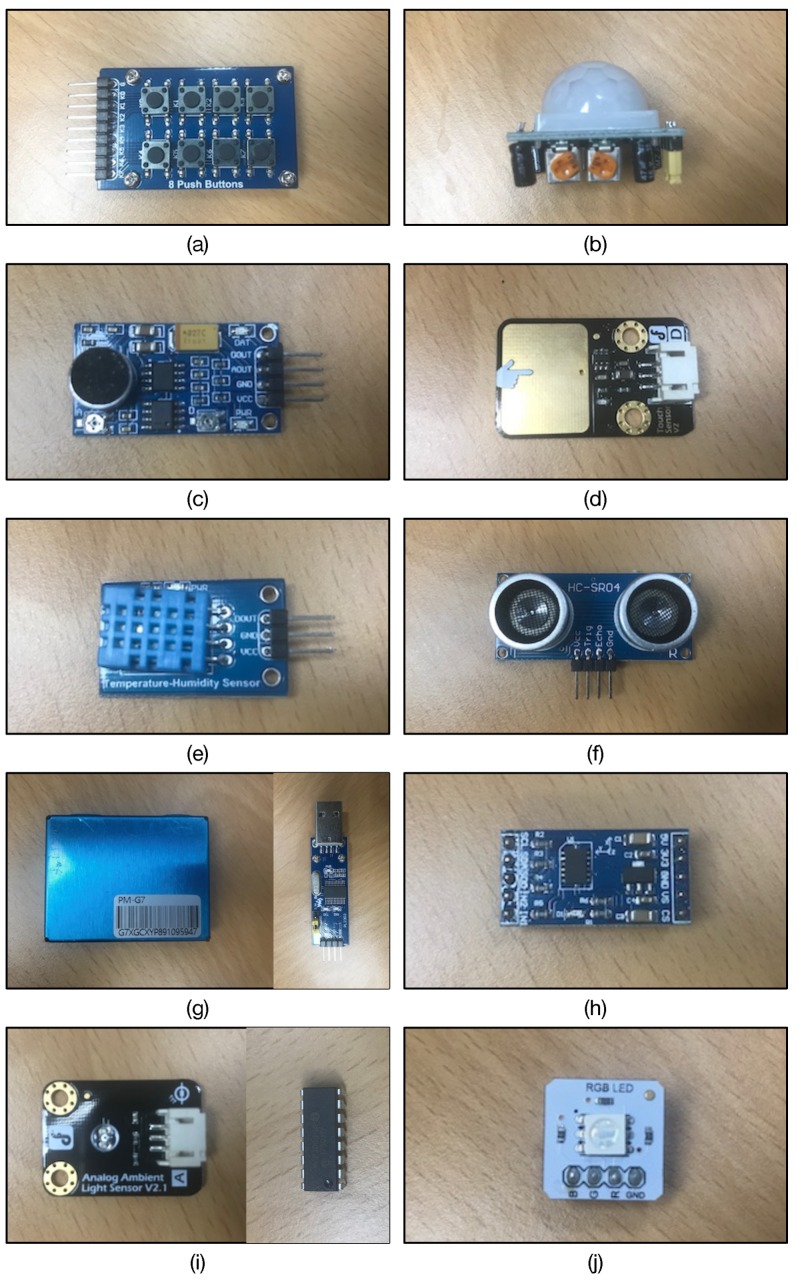
The photos of sensors and actuators deployed in our implementation work; (**a**) eight-pushbutton matrix, (**b**) passive infrared (PIR) sensor, (**c**) ambient sound sensor, (**d**) capacitive touch sensor, (**e**) temperature sensor, (**f**) ultrasonic sensor, (**g**) particulate matter sensor (left) with PL2303 USB-to-UART converter (right), (**h**) accelerometer, (**i**) ambient light sensor (left) with MCP3008 analog-to-digital converter (right), (**j**) RGB LED.

Finally, we have chosen ten types of hardware (nine sensors and one actuator) connected to the Raspberry Pi via the hardware interfaces listed in Table 2. Table 3 summarizes the hardware interfaces and Node.js modules (explained in the next section) for the sensors and actuator we have chosen. Figure 5 demonstrates the photos of sensors and actuator in our implementation work; (a) the eight-pushbutton matrix from WaveShare Electronics, (b) passive infrared (PIR) sensor (HC-SR501), (c) ambient sound sensor from WaveShare Electronics, (d) capacitive touch sensor from DFRobot, (e) temperature/humidity sensor (DHT11 module) from WaveShare Electronics, (f) ultrasonic sensor (HC-SR04P), (g) particulate matter (PM) sensor (PMS7003 from Plantower Technology) with USB-to-UART converter (PL2303 from WaveShare Electronics), (h) accelerometer (ADXL345 from Analog Devices embedded module), (i) ambient light sensor from DFRobot with analog-to-digital converter (MCP3008 from Microchips), (j) RGB LED module from DFRobot.

### 5.2. Implementation of the oneM2M Platforms and TAS

The Node.js source code for the Mobius, oneM2M server platform, is available in the OCEAN (Open allianCE for IoT stANdards), an open source-based global partnership project for IoT [45]. Developers can download all open-source software and codes for free and utilize them to develop IoT products and services under the BSD3-clause license. We can create our own IoT server based on the Mobius open-source. In this work, however, we employed the public Mobius-based IoT server providezd by Korea Electronics Technology Institute (KETI) for research and education. One advantage of using the KETI’s public IoT server is that we can use the Web-based oneM2M resource monitoring application that makes it easy to monitor sensing values and actuation commands of the IoT devices in real-time. Further details will be described in the next section.

The nCube, oneM2M middleware platform, has been developed using various open-source Node.js libraries, including express, fs, util, http, ip, url, coap, mqtt, websocket that are available in their GitHub repositories. The latest version of the nCube is available in the OCEAN Developer homepage [46]. The nCube is installed on the Raspberry Pi 3 Model B+ and configured to be registered with the KETI’s IoT server, i.e., create an AE for the Raspberry Pi under the CSE resource of the IoT server.

The most important part of implementing the nCube is building the TAS software programs for the sensors and actuator listed in Table 3, because they serve as the middleware programs translating sensing and actuation messages with the sensors and actuator. We developed the TAS programs using the widely used open-source Node.js libraries, including onoff for normal digital inputs and outputs [39], node-dht-sensor for DHT11 temperature sensor [40], pigpio for HC-SR04P ultrasonic sensor [41], serialport for UART communication [42], i2c-bus for I^2^C communication [43], spi-device for SPI communication [44], as shown in the rightmost column of Table 3. Of course, other Node.js libraries could be chosen and utilized to develop TAS programs supporting the hardware interfaces. All the TAS programs we have developed are available for free in the GitHub repository [47].

### 5.3. Resource Monitoring and Android Application

**Figure 6:**
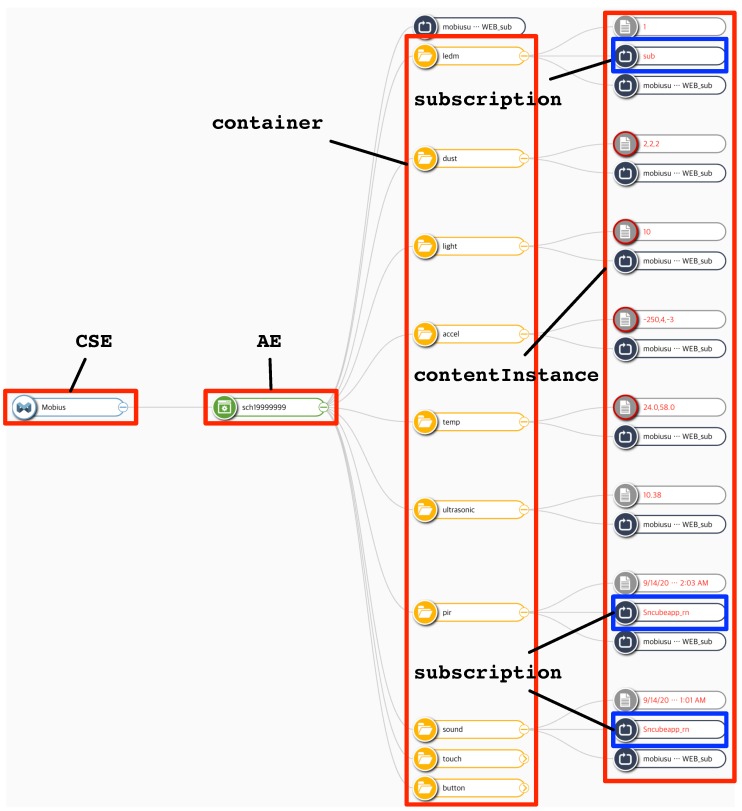
A captured image of the oneM2M resource monitor.

Once the nCube and TAS programs are installed in the Raspberry Pi, they start performing the functions of translating the sensing values into the corresponding oneM2M standard-based resources or vice versa in case of actuators. Figure 6 presents the captured image of the oneM2M resource monitor, which is only available in the public KETI’s IoT server. A similar standalone application (not Web-based) for resource monitoring is available in the OCEAN [48].

In Figure 6, we can see that the AE for the Raspberry Pi is created with the name of sch19999999 under the CSE resource for the Mobius. Subsequently, 10 containers are created under the AE resource, each of which is for the thing connected to the Raspberry Pi, i.e., nine sensors and one actuator. Here, we can say that a TAS program is responsible for creating its corresponding container. It should be noted that the rightmost column of the figure demonstrates the contentInstance and subscription resources under their parents containers. The topmost contentInstance under the ledm container is for the RGB LED actuator where ‘1’ stands for a control command such as ‘turn the red light on.’ Otherwise, the other contentInstance resources represent the string-formatted latest measured sensing values. For example, under the temp container, ‘24.0,58.0’ represents a temperature of 24° in Celsius and a relative humidity of 58%. The subscription resource under the ledm container is created by the Raspberry Pi (i.e., sch19999999) so that it could be notified when a new contentInstance is created under the ledm container (i.e., a new control command is triggered by other applications). The bottommost two subscription resources are created by the oneM2M application for Android (explained the next paragraph) so that it could be notified when new sensing values are updated in the pir and sound containers.

**Figure 7:**
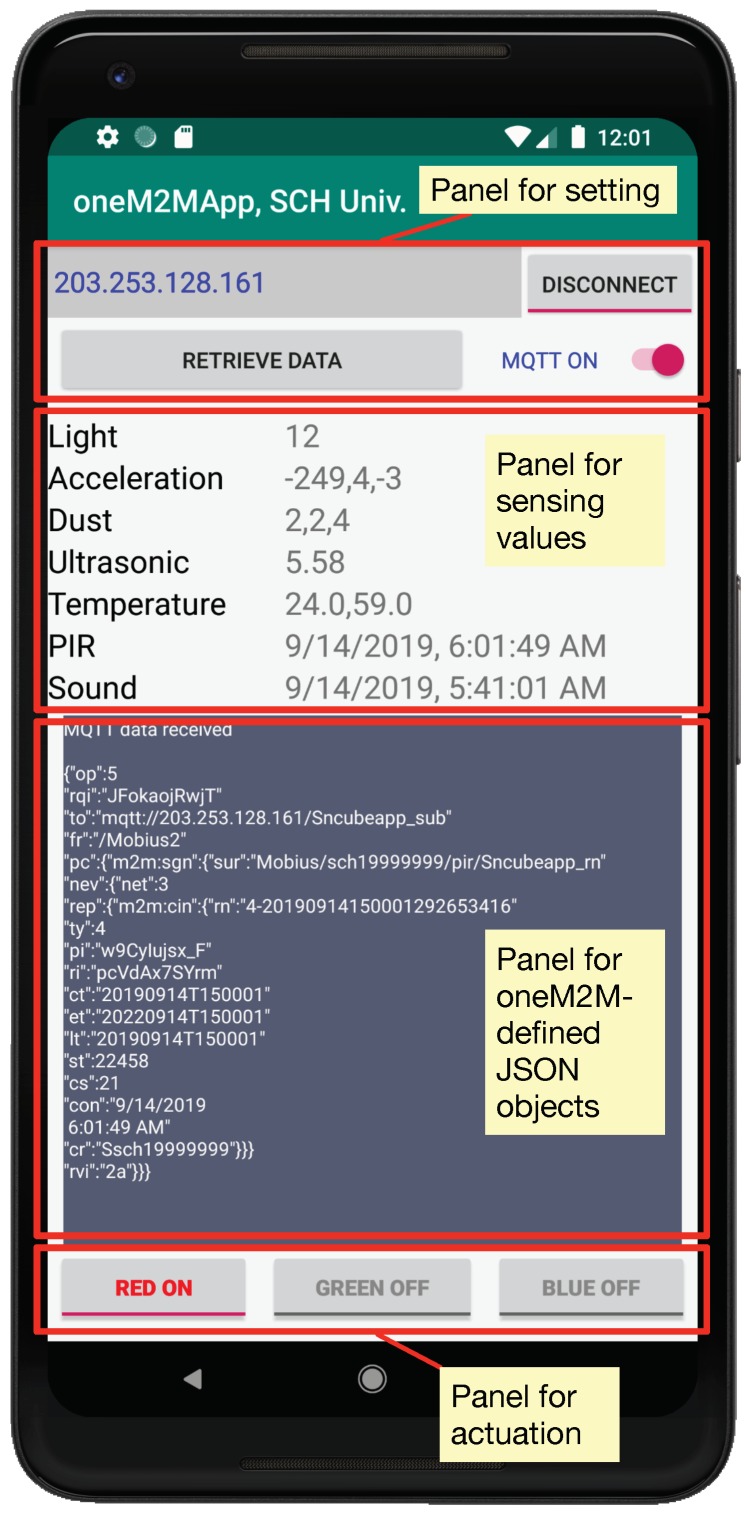
The captured image of the oneM2M Android application.

A smartphone might be the most user-friendly device interacting with people in our daily lives. Thus, a smartphone application working with the oneM2M platforms (i.e., Mobius and nCube) will help normal users to experience the benefits of standards-based IoT products and services. The OCEAN has already worked on an open-source project for developing an Android application working with the oneM2M platforms [49]. However, it only offers the TAS programs for digital output and UART communication interfaces. Accordingly, based on the open-source code in the OCEAN, we created a new Android application working with the Mobius and nCube, as shown in Figure 7. The Activity view mainly consists of four panels. The top panel is for setting the application, including the IoT server’s IP address, button for connecting the server, button for retrieving sensing values, sliding button for turning notification functions (i.e., MQTT) on. The second panel is for showing the measuring values of seven sensors (pushbutton and capacitive touch sensor are not considered in the application). Whereas the top five sensors can be retrieved by pressing the button named ‘Retrieve Data’ in the first panel, the time at which events are occurred in the PIR and ambient sound sensors can be notified when new contentInstance are created under their container, i.e., when someone is detected in a specified area of the PIR sensor and sound is detected around the space of the ambient sound sensor. The third panel is for presenting the oneM2M-defined JSON objects contained in HTTP messages from the Mobius, which can be used efficiently when debugging. The last one is for sending control commands to the RGB LED module, where the commands for turning the red light on and off are translated into 1 and 2, turning the green light on and off into 3 and 4, turning the blue light on and off into 5 and 6. The source code for the oneM2M Android application we have developed is also available for free in the GitHub repository [50].

## 6. Discussion and Remaining Challenges

The primary advantage of the proposed middleware platform is that it can provide sensing and actuation capabilities of IoT devices while complying with one of widely used IoT standards for the application service layer, oneM2M. IoT standards in the service layer like oneM2M focus on interworking of its service layer to underlying networks like 4G/5G networks or other service layer standards such as OCF (open connectivity foundation) or LwM2M (lightweight M2M) of OMA (open mobile alliance). It should be emphasized that integrating of its physical hardware interfaces to connected *things* (i.e., sensors and actuators) is also important to produce standards-based IoT devices. Unfortunately, however, most service layer standards including oneM2M leave parts of specification for integrating with legacy hardware interfaces of embedded systems such as UART unspecified, remaining a significant implementation issue for developers. We focus on this implementation issue and propose the oneM2M-based IoT middleware platform supporting the mostly used hardware interfaces in embedded systems including UART, I^2^C, and SPI, which enables developers to easily realize the commonly used sensing and actuation functions of IoT devices. It can also leverage the already-existing embedded systems to be integrated with the IoT systems newly constructed in the future.

Although the functional and non-functional requirements analyzed in the survey literature [3] (demonstrated in Table 1) are not comprehensively satisfied in the proposed IoT middleware platform, it is summarized in Table 4 what requirements we tried to take into consideration and how the requirements are realized in developing the oneM2M middleware platform. It should be also noted that some requirements are not fulfilled in the proposed platform and remains our future work.

### ***Functional requirements*** 

- Resource discovery:
  The oneM2M specifications define the common services provided by the application service layer in IoT systems, referred to as common service functions (CSFs) [51]. ‘Discovery’ is one of the oneM2M-defined CSFs which allows IoT entities to send discovery requests to search resources about applications and services.
- Resource management:
  The ‘resources’ considered in Table 1 include battery-time, memory usage, and other data related to application performance to make quality of service (QoS) reliable. Although some parts of this requirement rely on its implementation, the oneM2M CSFs of ‘Application and Service Layer Management’ and ‘Device Management’ could probably support these requirements.
- Data management:
  The oneM2M CSF of ‘Data Management and Repository’ is responsible for providing data storage and management converting aggregated data into a specific format and preparing for further analytics such as semantic processing.
- Event management:
  The oneM2M CSF of ‘Subscription and Notification’ can manage subscription to the resources hosted in the oneM2M platform, and can provide notification containing the changes on the resources to the address where the subscriber wants to receive them. Accordingly, application and services can acquire all the information about the proper events in real-time.
- Code management:
  The oneM2M CSF of ‘Device Management’ utilizes the already-existing technologies including broadband forum (BBF) TR-069, OMA-DM, LwM2M for managing device capabilities. Of course, code updating operations for IoT devices could be achieved with the help of management clients, servers, and adapters defined in the oneM2M CSF specifications.

### ***Non-functional requirements*** 

- Scalability:
  An IoT platform needs to support rapidly growing numbers of IoT devices and keep a certain level of QoS support. Although the scalability of an IoT platform is crucial, it highly depends on implementation and performance in IoT servers rather than connected devices. Accordingly, in support of a well-designed oneM2M-based IoT server we can say that our middleware platform may deliver some level of QoS appropriate for the given environment and applications.
- Real-time or timeliness:
  Our middleware platform is written in JavaScript and run on Node.js runtime environment built on Chrome’s V8 JavaScript engine. Because Node.js works in a single-thread, event-driven, non-blocking I/O model, it has shown powerful performance in building scalable network applications handling massive number of network connections simultaneously. Therefore, our proposed middleware platform written in Node.js would work well in real-time applications requiring simultaneous file access, network connection, or input and output peripherals.
- Availability:
  Availability could be achieved by ensuring some level of fault-tolerance. The developed IoT platform does not deal with all fault tolerance issues that mainly occur in hardware interfaces. However, a watchdog function is able to detect the failure of middleware components interacting with hardware interfaces, and restart or reconnect if needed.
- Security:
  Security is a very critical requirement in IoT solutions, and the oneM2M defines its security framework including identification, authorization and authentication. Our middleware platform can be registered to the oneM2M server (i.e., Mobius) as an application entity. It can attempt to access a list of authorized resources hosted by the server with its server-generated unique identifier and privileges, called access control policy. However, authentication and other security components such as certificates still remain incomplete.
- Privacy:
  A huge amount of data will be stored in IoT platforms, having great potential in providing people with valuable services across different application domains. At the same time, however, it leads to obvious privacy issues, for example, normal users obviously need to decide the level of disclosure for the collected personal data to protect privacy. Our current IoT platform provides no privacy-preserving solutions, but it would be possible to build a simple way by utilizing semantic descriptor resources defined oneM2M container resources that can describe their categories and impact in privacy concerns.
- Ease of deployment, maintenance, and use:
  The oneM2M CSF of ‘Device Management’ allows the application entities registered to an oneM2M server platform (i.e., our platform-installed devices) to be easily maintained through existing device management technologies. Also, the Node.js-based implementation enables the middleware components (i.e., Node.js modules) to be updated or replaced accordingly without any high-level of technical expertise.
- Interoperability:
  One of main advantages of the oneM2M standard is to provide oneM2M protocol bindings with most common service layer protocols including HTTP [52], CoAP [53], MQTT [54], and WebSocket [55], enabling interoperability and interworking in the application service layer. Also, it defines a standardized ontology-based interworking [56]. All the functions supported by our platform are exposed in the form of REST APIs, fostering collaboration in the domain of IoT.
- Spontaneous interaction:
  The oneM2M CSFs of ‘Registration’ and ‘Device Management’ are responsible for interacting with newly connected devices containing appropriate identifiers such as globally unique object identifiers (OIDs). However, more efforts are needed to realize a truly plug-and-play experience with minimal human intervention.
- Multiplicity:
  This requirement belongs to a part of intelligence for IoT devices, and the proposed IoT device platform provides no analytic tools on data or decision-making procedures depending on resource conditions, for example, recommending the most suitable (or currently available) one among multiple IoT devices offering the same service, which is one area of our future work.
- Adaptability and flexibility:
  Similar to the above requirement of multiplicity, we have not yet realized capabilities related to adaptability and flexibility, which allows platform-equipped devices to adapt themselves according to short-term or long-term changes in resource conditions, application scenarios, and surrounding environments, remaining our future work.

## 7. Conclusions

With the advent of IoT-enabling technologies, it is reasonable to expect that our environment such as home and workplace will be instrumented with a wide variety of IoT devices that probably rely on heterogeneous hardware systems and different underlying networks. In the highly fragmented IoT landscape, a software glue called IoT middleware plays a pivotal role in bridging the gap between different IoT systems, helping them talk to each other and work together in a collaborative manner. In particular, an IoT middleware solution should provide IoT devices with a translation function with which the measured data from sensors and control commands to actuators can be converted to the resources available in the Internet. Accordingly, we have proposed an oneM2M standard-based middleware platform for IoT devices, called nCube having sensing and actuation capabilities. The nCube enables sensing values and actuation commands to be translated into oneM2M resources accessible via REST APIs in oneM2M-defined standardized ways. All the source codes for the nCube, TAS (i.e., tiny middleware program for sensors and actuators), and Android application we have developed are available in the GitHub repositories. We hope our contribution will help other developers create new IoT products and applications that will enable to accessing each other in a standardized manner.

References

## Figures and Tables

**Table 1:** Functional and non-functional requirements for IoT middleware [3].

Functional Requirements	Non-Functional Requirements
Resource discovery	Scalability
Resource management	Real-time or timeliness
Data management	Availability
Event management	Security
Code management	Privacy
	Ease of deployment, maintenance, and use
	Interoperability
	Spontaneous interaction
	Multiplicity
	Adaptability and flexibility

**Table 2:** Hardware interfaces for the oneM2M middleware platform.

Types	Hardware Interfaces
I/O	Digital inputs and outputs
	UART (universal asynchronous receiver-transmitter)
Communication	SPI (serial peripheral interface)
	I^2^C (inter-integrated circuit)

**Table 3:** The sensors and actuators chosen for our implementation work and corresponding Node.js package deployed.

Types	Sensors and Actuators	Hardware Interfaces	Node.js Modules
	Pushbutton	Digital input	onoff [39]
	Passive infrared (PIR) sensor	Digital input	onoff [39]
	Ambient sound sensor	Digital input	onoff [39]
	Capacitive touch sensor	Digital input	onoff [39]
Sensors	Temperature sensor	Digital input	node-dht-sensor [40]
	Ultrasonic sensor	Digital input/output	pigpio [41]
	Particulate matter sensor	UART	serialport [42]
	Accelerometer	I^2^C	i2c-bus [43]
	Ambient light sensor (with ADC)	SPI	spi-device [44]
Actuators	RGB LED	Digital output	onoff [39]

**Table 4:** A summary of how the functional and non-functional requirements are realized in the proposed oneM2M-based middleware platform.

Type	Requirements	Support	Descriptions
	Resource discovery	◯	oneM2M supported
	Resource management	◯	oneM2M supported
Functional	Data management	◯	oneM2M supported
	Event management	◯	oneM2M supported
	Code management	◯	oneM2M supported
	Scalability	Δ	Implementation dependent
	Real-time or timeliness	◯	Node.js supported
	Availability	Δ	Partially realized
	Security	Δ	Partially realized
Non-functional	Privacy	×	Need to be developed
	Ease of deployment, maintenance, and use	◯	oneM2M/Node.js supported
	Interoperability	◯	oneM2M/Node.js supported
	Spontaneous interaction	Δ	Implementation dependent
	Multiplicity	×	Need to be developed
	Adaptability and flexibility	×	Need to be developed
